# Aortic elastic fiber degeneration during acute type a aortic dissection and reverse aortic remodeling

**DOI:** 10.1186/s13019-024-02577-2

**Published:** 2024-02-09

**Authors:** Trina Chen, Ivana Kholova, Timo Paavonen, Ari Mennander

**Affiliations:** 1 Tampere University Heart Hospital, Tampere University Medical School, SDSKIR, Elämänaukio 1, P.O. Box 2000, Tampere, FI-33521 Finland; 2 Department of Pathology, Fimlab Laboratories, Tampere University Hospital, Tampere University Medical School, Tampere, Finland

**Keywords:** Aortic elastic fiber degeneration, Acute type a aortic dissection, Aortic reoperation

## Abstract

**Background:**

Progression of proximal or distal aortic dilatation is defined as reverse aortic remodeling after surgery for acute type A aortic dissection (ATAAD) that may be dependent on aortic wall degeneration.

**Methods:**

We investigated whether aortic wall degeneration is associated with reverse aortic remodeling leading to aortic reoperation after surgery for ATAAD. Altogether, 141 consecutive patients undergoing surgery for ATAAD at Tampere were evaluated. The resected ascending aortic wall at surgery was processed for 42 degenerative, atherosclerotic and inflammatory histological variables. Patients undergoing aortic reoperations (Redos) were compared with those without aortic reoperations (Controls) during a mean 4.9-year follow-up.

**Results:**

Redos were younger than Controls (56 and 66 years, respectively, *P* < 0.001), and had less frequently previous cardiac surgery prior to ATAAD. Initial surgery encompassed replacement of the ascending aorta in the majority. There were 21 Redos in which one patient died during follow-up as compared with 51 deaths in Controls (log Rank *P* = 0.002). Histology of the aortic wall revealed increased elastic fiber fragmentation, loss, and disorganization in Redos as compared with Controls (2.1 ± 0.5 vs. 1.9 ± 0.5, Point score unit (PSU), *P* = 0.043 and 1.7 ± 0.8 vs. 1.2 ± 0.8, PSU, *P* = 0.016, respectively). Moderate atherosclerosis occurred less often in Redos vs. Controls (9.5% vs. 33%, PSU, *P* = 0.037, respectively).

**Conclusions:**

According to this exploratory study, histopathology reveals distinctive aortic wall degeneration during ATAAD. Reverse aortic remodeling after ATAAD is associated with the presence of ascending aortic wall elastic fiber fragmentation, loss and disorganization during ATAAD.

## Introduction

Acute type A aortic dissection (ATAAD) is a major emergency threat affecting 2% of the population with mortality reaching up to 45% without acute surgery [1]. The whole of the aorta may be affected by hemorrhagic and friable tissue that is vulnerable for rupture. Circulatory malperfusion, pericardial tamponade, strokes and comorbidities enhance risk of recovery, and even with acute surgery, mortality remains 10–25% [2]. Limited surgery is often contemplated as most suitable for salvage intervention, but residual aortic tissue left without surgery requires regular follow-up for possible reintervention [3]. Follow-up after surgery for ATAAD includes yearly imaging scanning of the aorta for indices of aortic instability such as aortic dilatation, pseudoaneurysms and increased aortic false lumen blood flow [4, 5] that determine reverse aortic remodeling and the need for aortic reoperation [6, 7]. However, the association of aortic wall degeneration with ATAAD and outcome after surgery remains controversial [8].

The Consensus statement on surgical pathology of the aorta from the Society for Cardiovascular Pathology and the Association for European Cardiovascular Pathology was recently released to clarify the nomenclature and diagnostic criteria of aortic degeneration [9]. The Consensus statement enables detailed means to investigate degenerative aortic wall changes pertinent to the development of ongoing aortic disease. As the aim of surveillance after salvage ATAAD surgery is the early identification of further aortic events, the aim of this study was to investigate the presence and significance of ascending aortic wall degeneration in ATAAD patients with or without reverse aortic remodeling requiring aortic reoperations in a single-center patient cohort.

## Methods

### Ethical statement and study protocol

After institutional review board approval (Ethical Committee of the Tampere University Hospital, Tampere, Finland, R15013), the need for informed consent was waived and the study conforms to the ethical guidelines of the Declaration of Helsinki. The ascending aortic wall resection of 141 consecutive patients undergoing surgery for ATAAD was obtained and processed for histology. All patients experienced onset of symptoms leading to surgery for ATAAD in less than 2 weeks. ATAAD was preoperatively confirmed and evaluated with computer tomography (CT) and transesophageal echocardiography, whenever possible. Surgery was performed between December 2008 and August 2021.

### Initial surgery

The decision on the extension of resection and surgical technique during ATAAD was at the discretion of the operating surgeon. When the aortic wall including the sinotubular junction (STJ) was estimated as the reason for aortic regurgitation, STJ was tailored for a suitable graft in a supracoronary fashion. Whenever ATAAD included the aorta root, a radical resection of the dissecting and dilated ascending aorta, together with the root and the aortic valve, was performed. Similarly, the aortic arch was either resected totally or in a hemiarch fashion, depending on the involvement of aortic wall disease. Intimal tears were resected whenever feasible. The graft size was estimated by the principal surgeon. Since the initial surgical procedure was performed upon surgical decision during ATAAD, the sample was procured from the middle of the resected area of the ascending aorta at the vicinity of STJ.

### Histology and immunohistochemistry

**Fig. 1:**
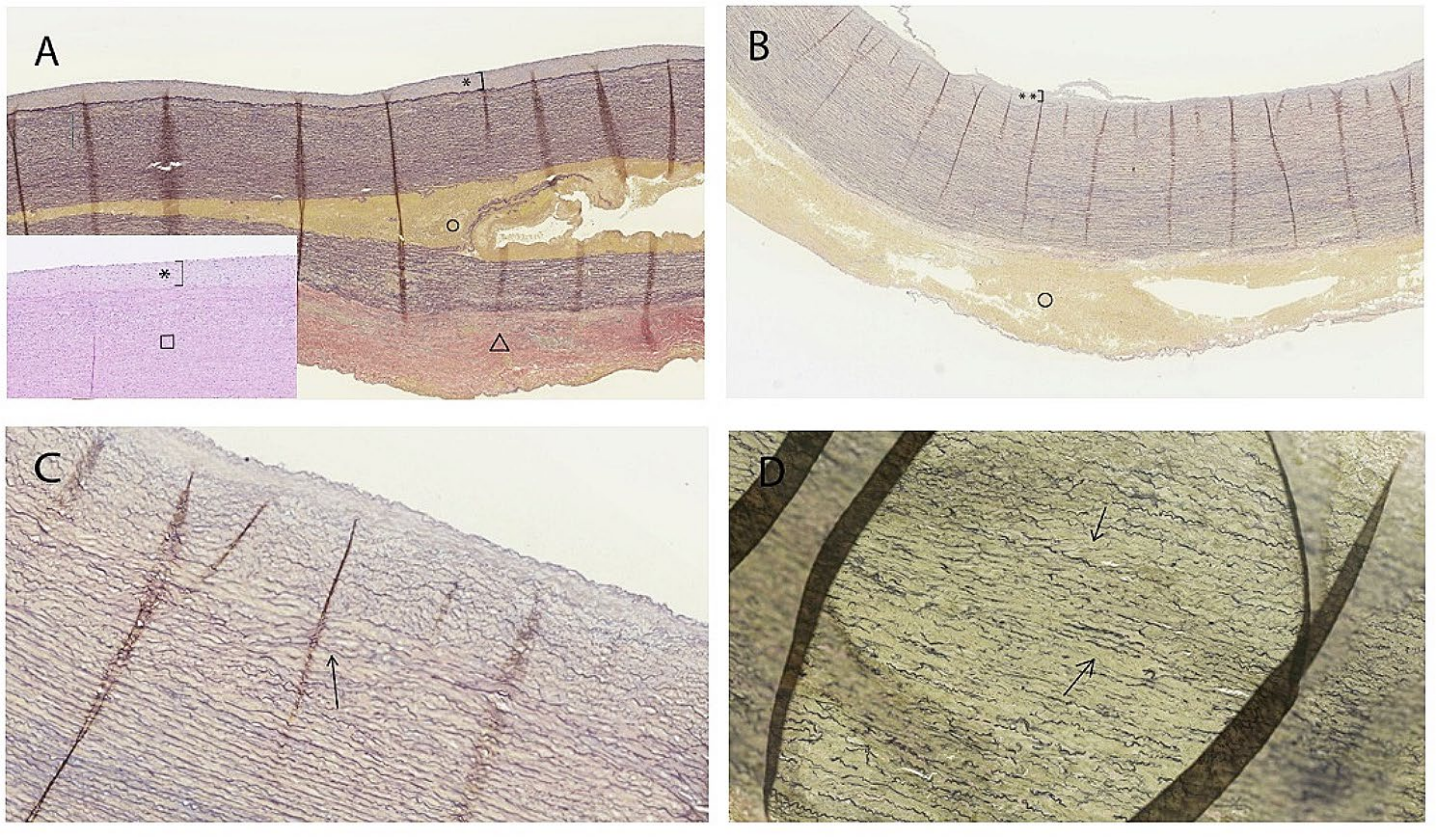
Aortic wall morphology in dissected ascending aorta. (**A**) Moderately expanded atherosclerotic intima (*). **Aortic** wall dissection in the outer third of the media layer (o). The adventitia is reactively thickened as reaction to media dissection (Δ). Verhoeff-Van Gieson, 100 x magnification. Haematoxylin-eosin, 100 x magnification (insert). (**B**) Mild atherosclerosis in the ascending aorta. Due to dissection in this aortic sample, only two thirds of the media are present in the section. Verhoeff-Van Gieson, 100 x magnification. (**C**) Detail of the media layer with thinned elastic fibers and focal disruption and loss of elastic fibers (arrow). Verhoeff-Van Gieson, 200 x magnification. (**D**) Severe loss and fragmentation of elastic fibers (arrows) Verhoeff-Van Gieson, 200 x magnification

Two to five blocks of resected ascending aorta including the adventitia, media and the intima, procured during the initial surgery for ATAAD, were embedded in paraffin, cut to 4 μm thick segments, and stained with Hematoxylin and Eosin, Verhoeff-van Gieson, Elastase-van Gieson and Periodic Acid-Schiff. A representative, one-cm long piece of ascending aortic wall corresponding to all different staining was evaluated systematically for all resected samples procured during surgery (Fig. 1).

Aortic wall histology and immunohistochemistry was performed using Ventana Lifesciences Benchmark XT© Staining module for leukocytes, T- and B-lymphocytes, plasma cells, macrophages, smooth muscle cells, cell proliferation, elastase and van Gieson staining. Ventana Lifesciences Antibody Dilution Buffer© was utilized for dilution media. The heights of different layers (adventitia, media and intima) were calculated for each sample [10].

### Quantification of medial degeneration

Medial degeneration of the ascending aorta was assessed by quantifying 11 different variables describing medial and adventitial damage [9, 11]. These included medial fibrosis, elastic fiber disorganization, elastic fiber loss/fragmentation, elastic fiber thinning, laminar medial collapse, classification of medial degeneration, mucoid extracellular matrix accumulation, smooth muscle cell disorganization, and smooth muscle cell nuclei loss and medial thickness of vasa vasorum, as well as adventitial fibrosis. According to consensus, the variables describing medial degeneration were categorized as none, mild, moderate and severe on a scale of 0–3 [9].

### Follow-up protocol

Documentation of mortality and morbidity was available for all patients. Follow-up consisted of physical examination, CT (computer tomography) and echocardiography at three months after surgery, and yearly CT thereafter. Mean follow-up for the patients was 4.9 years standard deviations (SD) 4. Decision for aortic reoperation included morbidity after initial surgery encompassing reverse aortic remodeling with need for proximal or distal aortic reoperation with an onset of new aortic dissection or rupture, or evidence of increasing aortic aneurysm observed by echocardiography or CT. According to our Institutional policy, aortic aneurysm included an aortic diameter more than 5.0-5.5 cm wide or aortic growth more than 1 cm in a year. This definition was adjusted to the presence of Marfan syndrome, sex, patient size and symptoms according to The Yale Center criteria [4].

### Statistical analysis

Continuous variables were expressed as means with SD, and were compared using the Mann-Whitney test. Categorical variables were presented as numbers and percentages, and were compared using χ^2^ or Fisher’s exact tests. In order to seek clinical relevance associated with immunohistochemistry at ATAAD surgery in an exploratory manner, the patients were divided into two groups in accordance with aortic reoperations, due to reverse aortic remodeling (Redos) or not (Controls) during follow-up. Unadjusted survival was evaluated by Kaplan-Meier analysis with log-rank tests. All analyses were conducted using the IBM SPSS Statistics version 26.0 (IBM Corporation, Armonk, NY, USA) with *P* < 0.05 as the criterion for significance.

## Results

### Patient characteristics at acute type a aortic dissection

Patient characteristics are shown in Table 1. There were 21 patients with subsequent Redos and 120 without need of additional surgery defined as Controls during follow-up. In total, there were 48 female patients (34.0%). The mean age for the patients with later Redos was 56 years (SD 14), while it was 66 years (SD 13) for Controls. None of the patients had vasculitis, and only two patients had arthritis, one in each group. Only seven patients had a known connective tissue disorder; two patients with subsequent Redos had Marfan syndrome and one had Loeys-Dietz. There were three Marfans and one patient with TGFbeta mutation among the Controls. Previous surgery such as coronary artery bypass grafting, aortic valve or aortic surgery were equally represented in both groups. Interestingly, the majority of all patients had no history of aortic dilatation prior to ATAAD (87.9%). Nine patients (6.4%) had bicuspid aortic valve. The mean aortic diameter was 54 mm (SD 11) and was available in only 26 patients before initial surgery for ATAAD. Altogether, the majority of all patients had aortic valve regurgitation (68.1%).

**Table 1:** Patient characteristics at acute type A aortic dissection

		All Patients		Redos		Controls		*P*-value
Number of patients		141		21		120		
Age, years (min, max)		64 (13)		56 (14)		66 (13)		< 0.001
Sex								
Female, n		48 (34.0%)		5		43		0.329
Male, n		93 (66.0%)		16		77		
Hypertension, n		83 (58.9%)		13		70		0.814
Diabetes, n		8 (5.7%)		0		8		0.605
Hypercholesterolemia, n		19 (13.5%)		2		17		0.739
Vasculitis, n		0 (0)		0		0		
Arthritis, n		2 (1.4%)		1		1		0.277
Asthma, n		10 (7.1%)		2		8		0.644
MCC, n		14 (9.9%)		0		14		0.129
Cerebrovascular stroke		3 (2.1%)		0		3		1
Earlier CABG, n		7 (5.0%)		0		7		0.594
Earlier AVR, n		7 (5.0%)		2		5		0.279
Earlier aneurysm, n		17 (12.1%)		2		15		1
Earlier aortic operation, n		9 (6.4%)		2		7		0.623
BAV, n [16]		9 (6.4%)		2		7		0.623
Connective tissue disorder, n		7 (4.2%)		3		4		0.068
AVI, n		96 (68.1%)		13		83		0.444

MCC = morbus coronaries cordis; CABG = coronary artery bypass grafting; AVR = aortic valve replacement; BAV = bicuspid aortic valve; AVI = aortic valve insufficiency

[16] = tricuspid aortic valve diagnosis according to reference #16

### Operative technique at acute type a aortic dissection

The initial operative technique for all patients is shown on Table 2. Slightly more than half of the patients (54.6%) had replacement of the ascending aorta only, while 15 patients required additional Redos. A conduit prosthesis including replacement of the aortic root together with an aortic valve prosthesis was required in only 54 patients, of which only five had subsequent Redos. An ascending aortic prosthesis together with aortic valve replacement, but without replacing the aortic root, was implanted in nine patients, and only one of these required subsequent Redos. Concomitant coronary artery bypass grafting was performed in 21 patients. There were two patients with early hemostasis, and one fasciotomy of the distal extremities.

**Table 2:** Initial surgery at acute type A aortic dissection

		All patients		Redos		Controls		*P*-value
		141		21		120		
Graft replacement of root and ascending aorta								
Mechanical conduit, n		15 (10.6%)		2 (9.5%)		13 (10.8%)		1
Biological conduit, n		39 (27.7%)		3 (14.3%)		36 (30.0%)		0.188
Graft replacement of ascending aorta								
Mechanical valve + prothesis, n		2 (1.4%)		0 (0)		2 (1.7%)		1
Biological valve + prothesis, n		7 (5.0%)		1 (4.8%)		6 (5.0%)		1
Prothesis, n		78 (54.6%)		15 (71.4%)		63 (53.0%)		0.153

### Perioperative findings, histology and immunohistochemistry at initial surgery during acute type a aortic dissection

As shown on Table 3, medial degeneration was characterized in all patients with ATAAD. Elastic fiber fragmentation and loss were increased in patients requiring subsequent Redos vs. Controls (2.1 ± 0.5 vs. 1.9 ± 0.5, *P* = 0.043, respectively). Similarly, the extent of elastic fiber disorganization was evident in Redos vs. not (and 1.7 ± 0.8 vs. 1.2 ± 0.8, *P* = 0.016, respectively). Only mild atherosclerotic features of the aortic wall were demonstrated in 52.4% patients with Redos vs. 28.3% without (*P* = 0.041, respectively), while moderate atherosclerosis was present in 9.5% Redos vs. 33.3% without (*P* = 0.037, respectively). The presence of granulomatous giant cell, lymphoplasmacytic, mixed inflammatory, or even suppurative patterns were present in some of the ATAAD patients, as a total group.

**Table 3:** Detailed histopathological evaluation/assessment according to the Society for Cardiovascular Pathology and the Association for European Cardiovascular Pathology guidelines (9)

		All patient		Redos		Controls		*P*-value
Overall Medial Degeneration, n		141		21		120		1
	Severity, mean (SD)	2.4 (0.6)		2.4 (0.5)		2.4 (0.6)		0.891
Mucoid Extracellular Matrix Accumulation, n		141 (100%)		21 (100%)		120 (100%)		1
	Extent, mean (SD)	2.0 (0.4)		2.0 (0.4)		2.0 (0.4)		0.800
	Severity, mean (SD)	2.0 (0.6)		2.0 (0.6)		2.0 (0.6)		0.908
Elastic Fiber Fragmentation And/Or Loss, n		141 (100%)		21 (100%)		120 (100%)		1
	Extent, mean (SD)	2.0 (0.5)		2.1(0.5)		1.9 (0.5)		0.043
	Severity, mean (SD)	2.0 (0.7)		2.1 (0.7)		1.9 (0.7)		0.348
Elastic Fiber Thinning, n		86 (61.0%)		15 (71.4%)		71 (59.2%)		0.339
	Extent, mean (SD)	1.0 (0.9)		1.2 (0.9)		1.0 (0.9)		0.301
	Severity, mean (SD)	0.9 (0.8)		1.2 (0.9)		0.9 (0.8)		0.091
Elastic Fiber Disorganization, n		112 (79.4%)		19 (90.5%)		93 (77.5%)		0.246
	Extent, mean (SD)	1.3 (0.8)		1.7 (0.8)		1.2 (0.8)		0.016
Smooth Muscle Cell Nuclei Loss, n		122 (86.5%)		19 (90.5%)		103 (85.8%)		0.739
	Type, mean (SD)	1.4 (0.7)		1.5 (0.7)		1.4 (0.7)		0.616
	Extent, mean (SD)	1.7 (0.9)		1.8 (0.9)		1.6 (0.9)		0.409
Laminal medial collapse, n		78 (55.3%)		11 (52.4%)		67 (55.8%)		0.815
	Type, mean (SD)	0.7 (0.7)		0.7 (0.7)		0.7 (0.7)		0.899
	Extent, mean (SD)	0.9 (0.9)		0.9 (1.0)		0.9 (0.9)		0.963
Smooth muscle cell disorganization, n		31 (22.0%)		7 (33.3%)		24 (20.0%)		0.250
	Extent, mean (SD)	0.3 (0.7)		0.6 (0.9)		0.3 (0.7)		0.134
Medial fibrosis, n		19 (13.5%)		4 (19.0%)		15 (12.5%)		0.486
	Extent, mean (SD)	0.3 (0.7)		0.3 (0.7)		0.3 (0.7)		0.458
	Severity, mean (SD)	0.3 (0.7)		0.3 (0.7)		0.3 (0.7)		0.455
Foreign body giant cell reaction, n		3 (2.1%)		1 (4.8%)		2 (1.7%)		0.386
Vaso vasorum medial thickening, n		22 (15.6%)		3 (14.3%)		19 (15.8%)		1
Adventitial fibrosis, n		26 (18.4%)		5 (23.8%)		21 (17.5%)		0.543
Atherosclerosis								
	Mild, n	45 (31.9%)		11 (52.4%)		34 (28.3%)		0.041
	Moderate, n	42 (29.8%)		2 (9.5%)		40 (33.3%)		0.037
	Severe, n	21 (14.9%)		2 (9.5%)		19 (15.8%)		0.740
	Atherosclerosis w/ Plaque Disruption and Surface Thrombus, n	1 (0.7%)		0 (0%)		1 (0.8%)		1
	Calcific atherosclerosis, n	9 (6.4%)		1 (4.8%)		8 (6.7%)		1
Inflammation								
Granulomatous/Giant cell pattern, n		5 (3.5%)		0 (0%)		5 (4.2%)		1
Lymphoplasmacytic pattern, n		24 (17.0%)		3 (14.3%)		21 (17.5%)		1
Mixed inflammatory pattern, n		3 (2.1%)		1 (4.8%)		2 (1.7%)		0.386
Suppurative pattern, n		5 (3.5%)		1 (4.8%)		4 (3.3%)		0.559

SD = standard deviation

### Aortic reoperations in Redos and survival

**Fig. 2:**
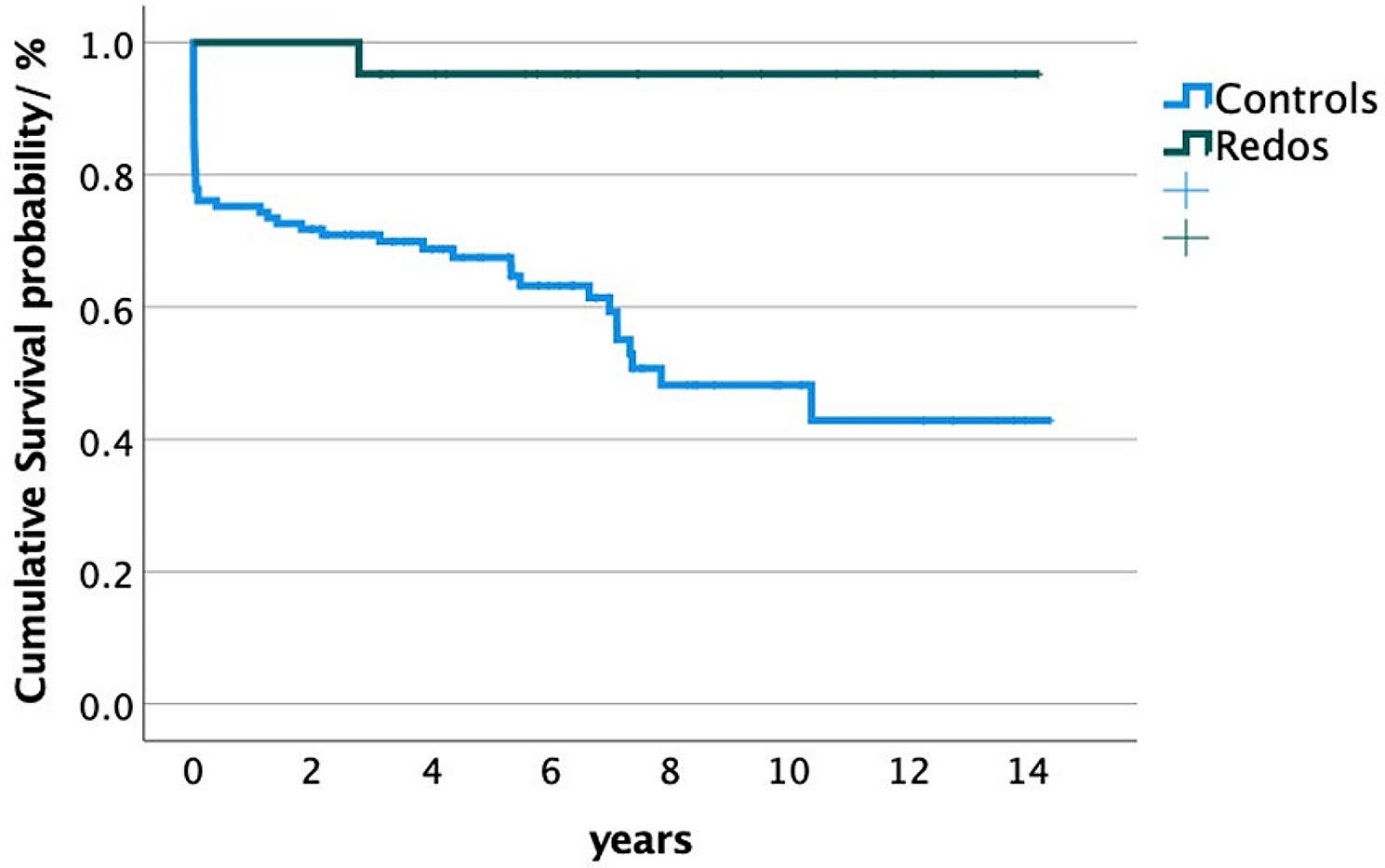
Survival probability (%) of patients after surgery for acute aortic type A dissection requiring aortic reoperations (Redos, green line*)* and without need of reoperations (Controls, blue line). Time-varying outcome according to Kaplan-Meier estimation. Log rank *P* = 0.002

In total, 21 patients required Redos. These included 18 patients reoperated due to the presence of an aneurysm. Only three patients required emergency Redos, all of which had an aortic root event including one aortic root dissection, one rupture, and one pseudoaneurysm (Table 4). Redos included six aortic root operations, of which three patients also underwent reconstruction of the distal aorta, including a frozen elephant trunk prosthesis. In addition, there were 15 patients that underwent Redos of the distal aorta including six endografts of the thoracoabdominal aorta, five frozen elephant trunk prosthesis, and four aortic arch reconstructions. Early 30-day mortality occurred in 31 of the 141 patients (22%). In total, there were 52 deaths among the patients, of which only one with Redos during follow-up (Fig. 2, log rank *P* = 0.002).

**Table 4:** Patients undergoing reoperations after initial surgery for Acute type A ascending aortic dissection

#	Age, years	Sex	Connective tissue disorder	Initial aortic surgery	Indications for redos	Urgency of redos	Time to redos, years	Redos/ surgery	Follow-up time, years	Extent of elastic fiber fragmentation and/or loss	Extent of elastic fiber disorganization	Moderate atherosclerosis
1	74	male		bioC + FET	descending aneurysm	elective	2.5	endograft	2.8	2	2	1
2	22	male	Loeys-Dietz	ascending + FET	descending aneurysm	elective	0.3	prosthesis	11.8	3	2	0
3	57	male		ascending + arch	descending aneurysm	elective	2.2	prosthesis	12.4	2	2	0
4	79	female		bioAVR + ascending	descending aneurysm	elective	0.2	endograft	10.8	2	1	0
5	48	female		mechC	arch aneurysm	elective	6.9	FET	9.6	2	1	0
6	58	male		ascending	AVI + root dissection	emergency	1.0	mechC	13.8	2	0	0
7	59	female		ascending	arch aneurysm	elective	7.2	prosthesis	7.5	2	2	0
8	64	female		ascending + hemiarch	root rupture+ arch aneurysm	emergency	0.3	bioC + FET	7.5	2	2	0
9	49	male		ascending + hemiarch	arch aneurysm	elective	0.5	FET	6.3	2	2	1
10	71	male		bioC	root pseudoaneurysm	emergency	1.4	bioC	6.3	2	1	0
11	56	male		ascending + arch	root aneurysm	elective	7.0	bioC	11.5	2	1	0
12	58	male		ascending	descending aneurysm	elective	0.2	endograft	8.9	2	2	0
13	62	male		bioC	arch aneurysm	elective	2.2	FET	3.4	3	3	0
14	39	male	Marfan	ascending + FET	descending aneurysm	elective	3.0	endograft	3.2	3	2	0
15	48	male		ascending + arch (MVR + ASD)	root aneurysm	elective	0.4	prosthesis (TVI)	3.2	3	3	0
16	57	male		ascending	arch aneurysm	elective	3.5	FET	4.1	2	2	0
17	53	male		ascending	root+ descending aneurysm + AVI	elective	11.6	bioC + FET	14.2	2	2	0
18	67	male		ascending + FET	descending aneurysm	elective	2.5	endograft	5.8	1	0	0
19	29	female	Marfan	mechC + FET	arch + descending aneurysm	elective	3.5	endograft	5.6	2	2	0
20	56	male		ascending	arch + descending aneurysm	elective	1.6	FET	4.3	2	2	0
21	61	male		ascending	AVI + descending true lumen collapse + arch aneurysm	elective	0.8	bioC + FET	6.5	2	2	0

redos = aortic reoperation; bioC = biological conduit; mechC = mechanical conduit; FET = frozen elephant trunk prosthesis; AVI = aortic valve insufficiency; bioAVR = biological aortic valve replacement; MVR = mechanical mitral valve replacement; ASD = atrial septum defect; TVI = tricuspid valve insufficiency

## Discussion

This exploratory study shows that degenerative ascending aortic wall elastic fiber fragmentation, loss and disorganization may be present during ATAAD. The Consensus statement on surgical pathology of the aorta from the Society for Cardiovascular Pathology and the Association for European Cardiovascular Pathology provide a valuable diagnostic platform to evaluate the degree of aortic wall degeneration [9].

Surgical correction of ATAAD is mandatory as soon as possible to decrease mortality and prevent irreversible morbidity [2]. As an extensive aortic resection during ATAAD increases the risk of early recovery due to technical challenges, such as long cardiopulmonary bypass time, a limited aortic repair strategy is often anticipated [12]. Since acute mortality even after emergent surgery for ATAAD exceeds 17%, major effort aims at ameliorating acute recovery [13]. A limited aortic resection and replacement is often appreciated, even during the clinical scenario including malperfusion, acute kidney insufficiency, presence of strokes and convulsions, pericardial tamponade and, least to mention, the longitudinally and horizontally ruptured aortic wall itself [12]. The surgical strategy of limited aortic resection during ATAAD is justified when vigilant follow-up and possible early intervention are offered to the patients. On the other hand, any extent of surgical aortic resection can scarcely ever preclude reverse aortic remodeling during patient recovery and follow-up of the remaining aorta or arterial anatomy [3, 5, 13].

Follow-up of the patients after surgery for ATAAD determines the need for reoperations [5]. Imaging techniques such as computer tomography performed regularly may reveal reverse aortic remodeling and signs of recurrent aortic events [4–6]. It is anticipated that the more aortic disease is left without replacement at initial ATAAD, the more potential risk is presented for recurrent ATAAD [3, 13]. In this study, the resected aortic wall was also investigated to compare various signs of aortic wall histopathology vs. reverse aortic remodeling. Though the Redos were younger than the Controls, the aortic wall sample revealed significant degenerative features as compared with the Controls.

A second phase of surgical intervention may be needed and includes resection of the diseased aortic tissue to prevent ongoing aortic events during follow-up. However, despite a limited resection of the ascending aorta during ATAAD, only approximately 11% of these patients may require reoperations [14], while the reoperation rate in this study was up to 15%. Interestingly, this study showed that survival of patients undergoing reoperations was not reduced as compared to patients without the need for reoperations. It is tempting to speculate that the ATAAD patient benefits from a surgical strategy that includes vigilant follow-up, understanding of the pathology of the aortic wall, and readiness for aortic reoperation whenever needed.

Aortic wall elastic fiber fragmentation, loss and disorganization, reflect and characterize degeneration of the aortic medial layer that may be prone to aortic events [15]. For diagnostic purposes, it would be essential to predict those patients with extensive risk of reverse aortic remodeling after ATAAD. The presence of these degenerative histological features may add to the planning of further resection of the residual aortic wall.

## Conclusions

Distinctive histopathological features of aortic wall degeneration during ATAAD reflect reverse aortic remodeling. Evaluating the quality of aortic degeneration during ATAAD may increase understanding of the pathogenesis of aortic dissection.

### Limitations

This pilot study represents a real-life single-center contemporary cohort. Though surgical techniques have evolved by time, we favored a tailored strategy for limited resection during ATAAD whenever possible [12]. The limitations of this study include the small number of patients with a relatively short follow-up, and aortic wall histology is obviously only available in patients that underwent surgery. Only all-cause mortality was available. During the emergency setting of surgery for ATAAD, procurement of a complete circular aortic sample is challenging; we did not report on the specific convexity or concavity sites of the aortic sample. The decision to undergo reoperation after initial surgery for ATAAD is dependent on radiological signs of reverse aortic remodeling. It would be valuable to compare aortic wall histopathology with the outcome in patients with ATAAD as a risk stratification model.

## Data Availability

The datasets used and analyzed during the current study are available from the corresponding author on reasonable request.

## Abbreviations

ATAAD: Acute type A aortic dissection
AVI: Aortic valve insufficiency
AVR: Aortic valve replacement
BAV: Bicuspid aortic valve
CABG: Coronary artery bypass graft
CT: Computer tomography
MCC: Morbus coronaries cordis
PSU: Point score unit
Redos: Aortic reoperations
SD: Standard deviation
STJ: Sinotubular junction
